# Corylin Confers Vasoprotection via JDP2‐Nrf2 Antioxidant Axis against Chronic Kidney Disease‐Induced Smooth Muscle Cell Remodelling

**DOI:** 10.1111/jcmm.71331

**Published:** 2026-08-20

**Authors:** Shang‐En Huang, Chih‐Le Lin, Kazunari K. Yokoyama, Kai‐Fang Hu, Bin‐Nan Wu, Zen‐Kong Dai, Meng‐Xuan Lin, Yu‐Hsin Tseng, Jong‐Hau Hsu, Jwu‐Lai Yeh

**Affiliations:** ^1^ Department of Seafood Science National Kaohsiung University of Science and Technology Kaohsiung Taiwan; ^2^ Department of Pharmacology, College of Medicine Kaohsiung Medical University Kaohsiung Taiwan; ^3^ School of Medicine, College of Medicine Kaohsiung Medical University Kaohsiung Taiwan; ^4^ Cell Therapy and Research Center Kaohsiung Medical University Hospital Kaohsiung Taiwan; ^5^ Graduated Institute of Medicine, College of Medicine Kaohsiung Medical University Kaohsiung Taiwan; ^6^ Department of Dentistry, Division of Periodontics Kaohsiung Medical University Hospital Kaohsiung Taiwan; ^7^ Department of Pediatrics Kaohsiung Medical University Hospital Kaohsiung Taiwan; ^8^ Department of Pediatrics, College of Medicine Kaohsiung Medical University Kaohsiung Taiwan; ^9^ Department of Medical Research Kaohsiung Medical University Hospital Kaohsiung Taiwan

**Keywords:** corylin, JDP2, oxidative stress, vascular calcification, VSMC phenotypic switching

## Abstract

Vascular calcification is a pathological process involving vascular smooth muscle cells (VSMCs) phenotypic switching into osteoblast‐like cells within a calcium phosphate‐deposited environment. This transformation is a critical risk factor for cardiovascular morbidity and mortality in patients with hyperphosphatemia due to chronic kidney disease. Corylin, a major compound derived from the Chinese medicine herb *Psoralea corylifolia* , is beneficial for the cardiovascular system. However, its role in modulating VSMC phenotypic switching and calcification remains to be elucidated. A vascular calcification rat model induced by vitamin D3 plus nicotine (VDN) was established to evaluate the therapeutic effects of corylin. The protective effects of corylin were evaluated in high phosphate‐induced vessels and VSMCs using cytotoxicity assay, Alizarin red S staining, alkaline phosphatase staining, immunofluorescence, Western blot analysis, and qPCR. The interaction between Jun dimerisation protein 2 (JDP2) and Nrf2 was analysed using a ChIP‐qPCR assay. Corylin treatment ameliorated arteriosclerotic lesions and oxidative stress in rats treated with VDN. We also observed that the cytoprotective effects of corylin prevented VSMC apoptosis and deposition of calcium phosphates. Additionally, corylin decreased calcium overload by inhibiting calcineurin and maintaining mitochondrial homeostasis. We identified a robust co‐induction of JDP2 and Nrf2 in corylin‐treated VSMCs, which contributed to reducing phenotypic switching and abrogating oxidative stress. Furthermore, Nrf2 knockdown by an inhibitor abolished JDP2 activation and the protective effects of corylin. Our study reveals that corylin exerts anti‐arteriosclerotic and vasculoprotective effects targeting JDP2‐Nrf2 activation, making it a potential treatment for vascular calcification.

## 1 Introduction

Chronic kidney disease (CKD) markedly increases the risk of cardiovascular morbidity and mortality, largely owing to vascular calcification, which is characterised by pathological mineral deposition within the vascular system, predominantly in the medial layer of the vessel wall [1]. Calcium phosphate deposition promotes structural damage, fibrosis, and calcium nodule formation in the arterial media, leading to arterial wall thickening and reduced vascular compliance [2, 3]. Unlike atherosclerosis, which is primarily characterised by intimal calcification, CKD predominantly induces medial arterial calcification through dysregulated calcium, phosphate, and vitamin D metabolism [4], oxidative stress [5], and osteogenic transdifferentiation of vascular smooth muscle cells (VSMCs), ultimately resulting in arterial stiffness and vascular remodelling [6].

The Jun dimerisation protein 2 (JDP2) is a member of the activating protein‐1 (AP‐1)/Activation transcription factor (ATF) family of transcription factors that dimerise with AP‐1/ATF to control the expression of genes like *AP1*/*ATF* [7]. In some cases, JDP2 can inhibit the activation of its binding partners, such as ATF2, indicating its role as a transcriptional repressor in epithelial‐like F9 cells [8]. JDP2 is a critical component of the nuclear factor‐erythroid 2‐related factor 2 (Nrf2)/small Maf protein family K (MafK) complex, which modulates reactive oxygen species (ROS) homeostasis, antioxidant‐ and senescence‐related programs [9, 10]. Moreover, in mouse embryonic fibroblasts, JDP2 associates with the MafK family transcription factors and Nrf2 to induce the antioxidant response element (ARE) component of various antioxidant‐related genes, inhibiting ROS production to maintain ROS homeostasis against oxidative stress at late stages in vitro [11]. Additionally, JDP2 overexpression has been shown to exert protective effects on neurological outcomes of mice with traumatic brain injury [12] and hypoxia‐reoxygenation‐induced cardiomyocyte injury [13]. Therefore, developing JDP2 modulators as therapeutic agents may provide a novel strategy for treating arteriosclerosis.

Corylin is a main flavonoid isolated from *Psoralea corylifolia* [14], a well‐known traditional medicinal plant used to treat various ailments since ancient times. An essential therapeutic component in Ayurveda and Chinese medicine, which is known for its antioxidant, antibacterial, and anticancer properties [15]. Recently, it has been reported that corylin increased Nrf2 activation and translocation, thereby preventing skin photoaging by reducing oxidative stress both in vitro and in vivo [16]. In addition, corylin reduces atherosclerosis in apolipoprotein E‐deficient mice fed a high‐cholesterol diet by inhibiting VSMC inflammation, proliferation, and migration [17]. To our knowledge, the mechanisms by which corylin moderates the osteogenic transformation of VSMCs have not been elucidated. Notably, whether corylin can target JDP2 to modulate vascular calcification remains elusive. Here, we investigated vascular and VSMC calcification using in vivo, ex vivo, and in vitro models. First, we investigated whether corylin treatment ameliorates vascular calcification and mineralisation in rats by administering vitamin D3 plus nicotine (VDN). Second, we assessed the potential of corylin to reduce high phosphate‐induced apoptosis, osteogenic differentiation, calcium overload, and oxidative stress by targeting JDP2 in ex vivo vessels and VSMCs.

## 2 Materials and Methods

### 2.1 Reagents

Corylin with a purity of ≥ 98% was purchased from ChemFaces Biochemical Co. (catalogue No. CFN98902, Wuhan, Hubei, China). Thiazolyl blue tetrazolium bromide (MTT) powder, sodium phosphate monobasic (NaH_2_PO_4_), sodium phosphate dibasic (Na_2_HPO_4_), ethylene glycol tetraacetic acid (EGTA), N‐acetylcysteine (NAC), and other reagent‐grade chemicals were purchased from Sigma‐Aldrich (St. Louis, MO, USA). QuantiChrom calcium and QuantiChrom alkaline phosphatase assay kits were purchased from Bioassay Systems (catalogue nos. #DICA‐500 and #DALP‐250; Hayward, CA, USA). Cellular calcineurin phosphatase activity assay kits were purchased from Abcam (catalogue no. ab139464; Waltham, MA, USA). Dulbecco's modified Eagle's medium (DMEM), fetal bovine serum (FBS), streptomycin, penicillin, and all other cell culture reagents were purchased from GIBCO BRL Life Technologies (Grand Island, NY, USA).

### 2.2 Experimental Animals

The specific pathogen‐free Sprague–Dawley (SD) rats were purchased from BioLasco (Taipei, Taiwan). The experiments complied with the ARRIVE guidelines 2.0 (conforming to the NIH Guide for the Care and Use of Laboratory Animals) and were approved by the Kaohsiung Medical University Animal Center (Institutional Animal Care and Use Committee approval no. 110006; Kaohsiung, Taiwan). All rats had free access to water and standard laboratory food throughout acclimatisation and experiments. Rats were housed in a well‐ventilated room under standard conditions (22°C ± 2°C, 60% ± 10% humidity) with a 12‐h light/dark cycle.

The model of aortic calcification produced by vitamin D_3_ plus nicotine (referred to as VDN) was developed following our previous study [18]. Thirty 6‐week‐old male SD rats (180–200 g) were randomly divided into the following five groups: A control group (CTL), a model group (VDN group), a corylin treatment group (VDN + Corylin), and the Corylin‐only groups (2 and 4 weeks). VDN and VDN + Corylin rats received 25 mg/kg nicotine in 5 mL corn oil (Sigma‐Aldrich), plus intramuscular injection of vitamin D_3_ (3 × 10^5^ IU/kg). Subsequently, VDN + Corylin and Corylin‐only rats received corylin (25 mg/kg i.p.) every 2 days, while CTL and VDN rats received an equal volume of solvent. The corylin dosage was based on an earlier study incorporating minor adjustments [19]. After four weeks of treatment, all rats were euthanised by CO_2_ asphyxiation, ensuring respiratory cessation, followed by decapitation. Thoracic aortic tissues were collected and frozen at −80°C for molecular analysis or fixed in 10% neutral‐buffered formalin for histological examination.

### 2.3 *Ex vivo* Culture

Thoracic aortas were harvested from healthy male SD rats (*n* = 20, 6 weeks old, 180–200 g). The adjacent connective tissue was gently removed, and the aortas were washed with phosphate‐buffered saline (PBS). The aorta was divided into five pieces and cultured in 24‐well plates with DMEM supplemented with 20% FBS and 1% penicillin/streptomycin (GIBCO) at 37°C in a humidified atmosphere with 5% CO_2_.

### 2.4 Cell Culture

Primary rat vascular smooth muscle cells (VSMCs) were obtained from the thoracic aorta of twenty 6‐week‐old male SD rats as described previously [20]. VSMC culture purity was confirmed by α‐SMA immunocytochemistry. Rat VSMCs were cultured in DMEM with 10% FBS and 1% penicillin/streptomycin (GIBCO), while human aortic SMCs (catalogue no. C‐007‐5C, Cascade Biologics, Portland, OR, USA) were cultured in Medium 231 with a 1% smooth muscle growth supplement following our previous study [21]. These VSMCs were incubated at 37°C with 5% CO_2_, with media refreshed every 3 days. VSMCs were treated with normal (0.9 mM) or high (3.0 mM) inorganic phosphate (NaH_2_PO_4_:Na_2_HPO_4_ = 1:2, pH = 7.0) as in previous studies [22, 23], and corylin was added 1 h before high inorganic phosphate (Pi) stimulation.

### 2.5 Histopathological Examination

Aortic tissues were fixed with 10% paraformaldehyde, embedded in paraffin, and cut into 5 μm‐thick sections. Then, they were dewaxed with xylene, hydrated through graded ethanol, and stained with hematoxylin (Sigma‐Aldrich) for 1 min, followed by staining with eosin (Sigma‐Aldrich) for 3 min. After dehydration and xylene rinsing, tissues were carefully mounted beneath a cover slip and observed under an optical microscope (Eclipse TE2000‐S; Nikon, Tokyo, Japan). The original images were used to evaluate the diameters of the tunica media using AxioVision Rel 4.6 software (Carl Zeiss, Oberkochen, Germany). The measurements of medial thickness were averaged across at least three locations randomly selected from each slice [24].

### 2.6 Alizarin Red S Staining

Aortic tissues obtained from the rats were embedded, dewaxed, and hydrated with alcohol. Afterwards, aortic sections were stained with 2% Alizarin Red S solution (Sigma‐Aldrich) for 30 min at room temperature. Any unbound stain was removed by washing with distilled water, and the slides were photographed under the microscope (Eclipse TE2000‐S; Nikon).

Cultured VSMCs were fixed with 4% paraformaldehyde for 30 min, washed with PBS, and stained with 2% Alizarin red solution for 30 min. After obtaining micrograph images of the VSMCs through a microscope, the staining was quantified using cetylpyridinium chloride extraction (Sigma‐Aldrich). The calcified mineral content was determined using an ELISA plate‐reading spectrophotometer (DYNEX Technologies, Germany) at 405 nm.

### 2.7 Measurement of Calcium Content

To quantify the calcium content in the aortic tissues, the tissues were homogenised and prepared in lysis buffer (20 mM Tris–HCl, 1 mM dithiothreitol, 5 mM EGTA, 2 mM EDTA, 0.5 mM PMSF, 20 μM leupeptin, and 20 μM aprotinin). The calcium content was measured by a calcium assay kit (catalogue no. #DICA‐500, BioAssay Systems) according to the manufacturer's instructions. The absorbance was measured using an ELISA‐plate reading spectrophotometer (DYNEX Technologies) at 612 nm.

### 2.8 Measurement of Alkaline Phosphatase Activity

Aortic tissues and VSMCs were homogenised in 0.1 M NaHCO_3_‐Na_2_CO_3_ buffer (pH = 9) containing 0.1% Triton X‐100, 2 mM MgSO_4_, and 6 mM 4‐nitrophenyl phosphate for 1 h at room temperature. After centrifuging, the supernatant was tested for protein content and alkaline phosphatase activity (#DALP‐500, BioAssay Systems) following the manufacturer's instructions. The protein content of the samples was quantified using a Pierce BCA Protein Assay kit (A55864, Thermo Scientific, Waltham, MA, USA). The absorbances in the enzyme assay were measured using an ELISA‐plate reading spectrophotometer (DYNEX Technologies) at 405 nm.

### 2.9 Immunofluorescence Staining

Thoracic rat aorta tissues were immersed in saccharose (30% w/v), embedded in optimal cutting temperature (OCT), and stored at −20°C until immunofluorescence analysis. Samples were cryosectioned (10 μm‐thick), mounted on glass slides, and fixed with 10% paraformaldehyde. After being washed, nonspecific binding sites were blocked for 30 min with 5% BSA and then incubated with rabbit antibodies against BMP‐2 (ARG57829, Arigo ARG57829, 1:100; Arigo Biolaboratories, Zhubei City, Taiwan) and α‐SMA (#19245, 1:100; Cell Signalling, Danvers, MA, USA) overnight at 4°C. Thereafter, the sections were incubated with a goat anti‐Rabbit IgG secondary antibody, (H + L)‐FITC conjugate (AP307F, 1:100; Sigma‐Aldrich), and the nuclei were stained with DAPI (Sigma‐Aldrich) at room temperature.

Rat VSMCs were seeded on microscope slides in 6‐well plates (10^4^ cells/well). After high Pi induction and with or without corylin treatment, cells were incubated with 4% paraformaldehyde, and nonspecific binding sites were blocked for 30 min with 5% BSA. Slides were incubated with rabbit antibody against Caspase‐3 (#9662, 1:100; Cell Signaling) and JDP2 (ab40916, 1:100; Abcam) overnight at 4°C. Subsequently, the sections were incubated with the Cy3‐ and FITC‐conjugated goat anti‐rabbit IgG secondary antibodies, respectively. Rhodamine‐conjugated phalloidin stained VSMC morphology, while DAPI stained nuclei. Micrographs were acquired using a confocal laser‐scanning microscope (Fluoview FV1000; Olympus, Tokyo, Japan), and fluorescence intensity was quantified using ImageJ 1.44 software (National Institutes of Health, Bethesda, USA).

### 2.10 Reactive Oxygen Species Detection

Rat tissue from the thoracic aorta was immersed in saccharose (30%) and embedded in OCT at −20°C. Subsequently, frozen sections (10 μm) of thoracic aortas were mounted on poly‐L‐lysine‐coated glass slides and incubated in 4 μM dihydroethidium (DHE; Thermo Fisher Scientific, Waltham, MA, USA) for 30 min at 37°C. The slides were washed with PBS, mounted, and visualised by the confocal laser‐scanning microscope (Fluoview FV1000; Olympus).

VSMCs were seeded into 96‐well plates at 10^3^ cells/well. After treatment with high Pi, with or without corylin, the cells were washed with PBS and incubated with 10 μM DHE or 2,7‐dichlorodihydrofluorescein diacetate (DCFH‐DA; Thermo Fisher Scientific) for 30 min, respectively. The cells were visualised by the fluorescence microscope (TE2000‐S; Nikon). The fluorescence intensity was quantified by ImageJ (National Institutes of Health).

### 2.11 Measurement of Cell Viability

VSMCs were incubated in 96‐well plates. After pretreatment with various concentrations of corylin, a high Pi stimulus was applied. Subsequently, the culture medium was replaced with MTT‐thiazolyl blue tetrazolium solution (0.5 mg/mL) for 2 h at 37°C. After the excess MTT solution was discarded, DMSO was added to each well for 15 min. The absorbance of MTT formazan crystals was detected using an ELISA‐plate reading spectrophotometer (DYNEX Technologies) at 540 and 630 nm.

### 2.12 Measurement of Intracellular Calcium Activation and Calcineurin Activity

VSMCs were incubated in Ca^2+^‐free HBSS at 37°C with 5% CO_2_ in 96‐well plates. After corylin pretreatment and high Pi for 1 day, Cal‐520 AM dye (2 μM, ab171868; Abcam) was added. Following 60 min incubation, the cells were visualised using fluorescence microscopy (TE2000‐S; Nikon), and intracellular calcium fluorescence intensity was quantified using ImageJ (National Institutes of Health). Calcineurin activity was assayed using a cellular calcineurin phosphatase activity assay kit (ab139464; Abcam) according to the manufacturer's instructions, with absorbance detected using an ELISA‐plate reading spectrophotometer (DYNEX Technologies) at 620 nm.

### 2.13 Mitochondrial and Nuclear Isolation from Cells

VSMCs were seeded in the 6 cm dishes; after corylin pretreatment and stimulation with high Pi for 1 day, the cells were harvested and centrifuged at 559 x g for 5 min. Mitochondrial proteins were isolated using a mitochondria isolation kit (catalogue no. #89874; Thermo Scientific) according to the manufacturer's instructions. The nuclear protein was isolated using a NE‐PER nuclear and cytoplasmic extraction kit (catalogue no. #78833; Thermo Scientific). These mitochondrial and nuclear protein expression levels were assayed by Western blotting.

### 2.14 Western Blot Assay

The cell extracts were prepared with lysis buffer containing 20 mM Tris–HCl (pH = 7.5), 1 mM dithiothreitol, 5 mM EGTA, 2 mM EDTA, 0.5 mM PMSF, 20 μM leupeptin, and 20 μM aprotinin. The cell lysate was centrifuged at 21,836 x g for 30 min, and the supernatant fraction was collected for Western blotting analysis. The protein content of the samples was quantified using a Pierce BCA Protein Assay kit (A55864, Thermo Scientific). Equivalent amounts of protein were subjected to SDS‐polyacrylamide electrophoresis gels (10 or 12%) and then transferred to polyvinylidene difluoride (PVDF) membranes. Nonspecific binding sites on the membranes were blocked with 5% skim milk in Tris‐buffered saline for 1 h and incubated overnight at 4°C with the following primary antibodies, as listed in Table 1. Thereafter, the blots were incubated with suitably buffered solutions of appropriate HRP‐linked secondary antibodies for 1 h at room temperature and visualised using an enhanced chemiluminescence (ECL) system (Merck Millipore, Burlington, MA, USA). The images were captured with a charge‐coupled device camera (MiniChemi‐Chemiluminescence; Sage Creation Sciences, BioRiver, Beijing, China). All of the immunoreactive bands were quantified using ImageJ (National Institutes of Health).

**TABLE 1:** List of antibodies utilised in this study.

Antibody and species	Catalogue number and dilution
Rabbit polyclonal anti‐Bax	GTX109683, 1:1000; GeneTex, Zeeland, MI, USA
Mouse monoclonal anti‐Bcl‐2	610,539, 1:1000; BD Bioscience, Franklin Lakes, NJ, USA
Rabbit polyclonal anti‐Cytochrome C	GTX108585, 1:1000; GeneTex, Zeeland, MI, USA
Rabbit polyclonal anti‐Caspase‐3	#9662, 1:1000; Cell Signalling, Danvers, MA, USA
Rabbit polyclonal anti‐PARP	GTX100573,1:1000; GeneTex
Rabbit polyclonal anti‐BMP‐2	ARG57829, 1:500; Arigo Biolaboratories, Zhubei City, Taiwan
Mouse monoclonal anti‐Runx2	sc‐390,351, 1:500; Santa Cruz Biotechnology, Dallas, TX, USA
Rabbit monoclonal anti‐α‐SMA	#19245, 1:1000; Cell Signaling
Rabbit polyclonal anti‐SM22α	ARG57892, 1:1000; Arigo Biolaboratories
Rabbit polyclonal anti‐Drp1	#8570, 1:1000; Cell Signaling
Rabbit polyclonal anti‐OPA1	ab42364, 1:1000; Abcam, Waltham, MA, USA
Rabbit polyclonal anti‐Mfn2	ab50838, 1:1000; Abcam
Rabbit monoclonal anti‐phospho‐c‐jun [Ser63]	NBP3‐21583, 1:1000; Novus Biologicals, Littleton, CO, USA
Rabbit polyclonal anti‐c‐jun	NB600‐1482, 1:1000; Novus Biologicals
Rabbit polyclonal anti‐JDP2	ab40916, 1:1000; Abcam
Rabbit polyclonal anti‐NFE2L2	TCBA 1560, 1:1000; Taiclone, Taipei, Taiwan
Rabbit polyclonal anti‐SOD1	GTX100554, 1:1000; GeneTex
Rabbit polyclonal anti‐SOD2	GTX116093, 1:1000; GeneTex
Mouse monoclonal anti‐beta‐actin	GTX629630, 1:2000; GeneTex
Rabbit polyclonal anti‐VDAC	#4661, 1:1000; Cell Signaling

### 2.15 ChIP‐Quantitative Real‐Time PCR

The ChIP assay was performed as described previously [11] with some modifications by using a SimpleChIP Enzymatic Chromatin IP kit (#9003; Cell Signalling Technology, Danvers, MA, USA) according to the manufacturer's instructions. In brief, corylin‐treated human VSMCs were incubated with 1% formaldehyde for 10 min and then sonicated to generate chromatin fragments at room temperature. The chromatin fragments were subjected to a ChIP assay using a rabbit polyclonal anti‐Nrf2 antibody (PA5‐27882, 5 μg; Thermo Fisher Scientific) or IgG antibody (#2729; 5 μg; Cell Signalling) and incubated overnight at 4°C. The DNA‐protein complexes were washed and precipitated using Protein A Sepharose beads incubated for 2 h at 4°C. The immunoprecipitated DNA was decrosslinked, and the DNA samples were purified using a phenol‐chloroform‐isoamyl alcohol solution and then analysed by quantitative real‐time PCR using the *Jdp2* primer sequence as described in the following:

### 2.16 Real‐Time Reverse Transcription PCR

Total RNA was extracted from rat tissues and human VSMCs using the SV Total RNA Isolation System (catalogue no. #Z3101; Promega, Madison, WI, USA) according to the manufacturer's instructions. We reverse‐transcribed 0.5 mg of total RNA samples using a Reverse Transcription System kit (catalogue no. #A3500; Promega). The PCR mixture was prepared using 40 ng cDNA, GoTaq qPCR Master Mix with SYBR Green and Low ROX dyes, and 0.5 μM primers, and then amplified with the specific primer pairs. Rapid cycle DNA amplification was performed as described previously [11], with thermocycling conditions as follows: Initial denaturation at 95°C for 10 min, followed by 40 cycles of 95°C for 15 s and annealing at 60°C for 60 s. The sequences of primers synthesised by Omicsbio Biotechnology Co. Ltd. (Taiwan) were as follows: Rat *ALP* (forward, 5′‐GTC ACA GCC AGT CCC TCA AC‐3′ and reverse, 5′‐TAT TCC AAA CAG GGG AGT CG‐3′), rat *BMP‐2* (forward, 5′‐GAC ATC CAC TCC ACA AAC GAG‐3′ and reverse, 5′‐GTC ATT CCA CCC CAC ATC ACT‐3′), rat *α‐SMA* (forward, 5′‐CAT TGC TGA CAG GAT GCA GAA‐3′ and reverse, 5′‐CGC CGA TCC AGA CAG AAT ATT T‐3′), and rat *GAPDH* (forward, 5′‐AAC TCC CAT TCC TCC ACC TT‐3′ and reverse, 5′‐GAG GGC CTC TCT CTT GCT CT‐3′), human *Jdp2* (forward, 5′‐CGG GGG ACT GCA TGG AAC‐3′ and reverse, 5′‐CCA GGC ATC ATA GCA GGA‐3′), human *BMP‐2* (forward, 5′‐GCG TGA AAA GAG AGA CTG CG‐3′ and reverse, 5′‐ACC ATG GTC GAC CTT TAG GA‐3′), human *α‐SMA* (forward, 5′‐GCT GTT TTC CCA TCC ATT GTG‐3′ and reverse, 5′‐GGG TCA GGA TTC CTC TTT TGC‐3′), and human *HPRT* (forward, 5′‐TCT TTG CTG ACC TGC TGG ATT ACA‐3′ and reverse, 5′‐AGT TGA GAG ATC ATC TCC ACC AAT‐3′). Real‐time PCR was performed on an ABI StepOnePlus System (Applied Biosystems, Foster City, CA) using Power SYBR Green PCR Master Mix (Takara Bio, Otsu, Japan). Gene expression was normalised with rat *GAPDH* or human *HPRT* as an internal control using the 2^−∆∆Ct^ method [11, 21].

### 2.17 Statistical Analysis

All continuous variables were expressed as mean ± standard error of the mean (SEM) for at least five replicates. An unpaired two‐tailed Student *t*‐test was applied for two group comparisons. Three or more groups were compared using one‐way ANOVA to determine if there were significant differences, followed by post hoc analysis (Bonferroni test) to identify specific group differences. *p* < 0.05 was considered to indicate a statistically significant difference. The analyses were conducted using GraphPad Prism software (version 5.0; GraphPad, La Jolla, CA, USA).

## 3 Results

### 3.1 Effects of Corylin on Aortic Calcification

**FIGURE 1:**
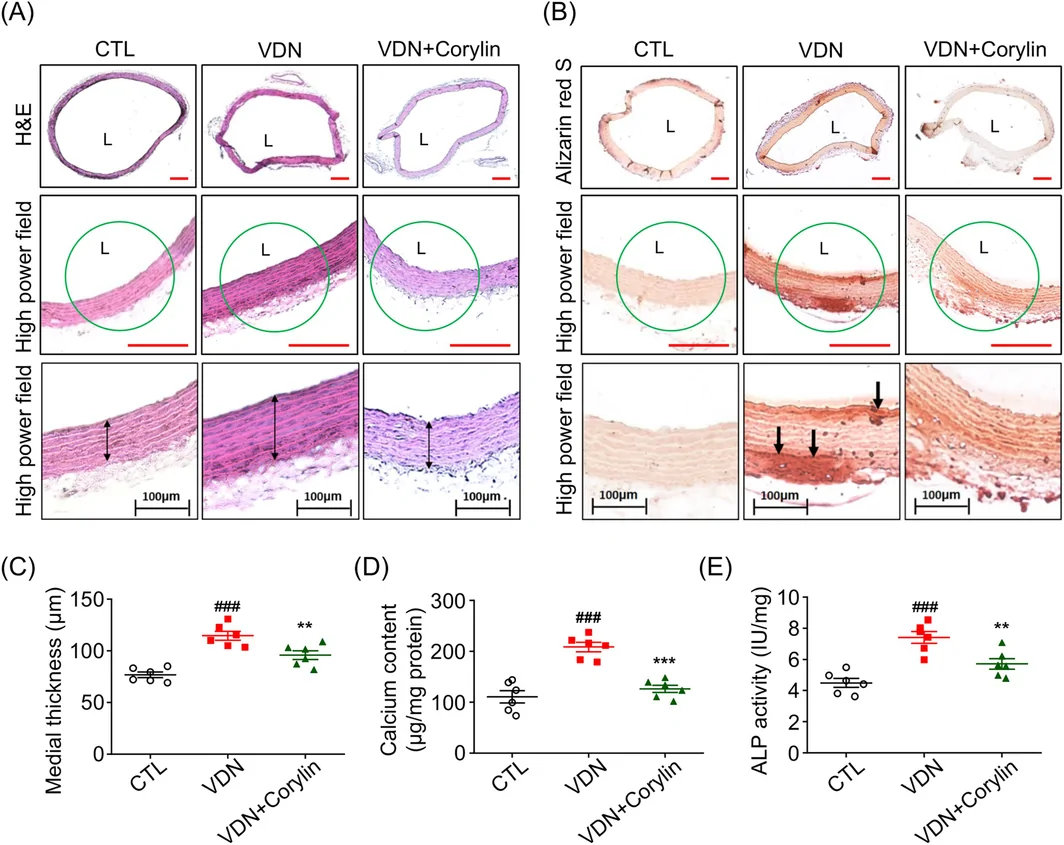
Corylin ameliorates aortic calcification in rats by nicotine plus vitamin D3 (VDN). (A) Representative histological images with hematoxylin and eosin (H&E) staining. (B) The histological assay of calcium by Alizarin red S staining. (C) The thickness of the tunica media was quantified using AxioVision Rel 4.6 software. (D) Quantification of the calcium content and (E) alkaline phosphatase (ALP) activity in the thoracic aorta homogenates. L indicates the lumen. The red scale bar indicates 200 μm, and the black scale bar indicates 100 μm. One‐way ANOVA was used for multiple‐group comparisons. All values are presented as mean ± SEM, *n* = 6. ^###^ *p* < 0.001 compared with the untreated control group. **p < 0.01 and ***p < 0.001 compared with the VDN‐treated group.

An animal model of vascular calcification induced in rats by vitamin D3 plus nicotine (VDN) administration was used to simulate patients with vascular calcification in vivo [18, 20, 25]. This model is closely similar to human vascular calcification related to ageing and pathologies, such as CKD and end‐stage renal failure [26]. We measured blood urea nitrogen (BUN) and creatinine (CRE) levels to evaluate renal function and to confirm the effects of VDN on the kidneys, as shown in Table 2. The effects of corylin treatment (25 mg/kg) were thus evaluated in vivo using this VDN rat model. After 4 weeks, VDN rats had thicker medial vessel walls compared to controls, and corylin treatment significantly decreased this thickness, as assessed by H & E staining (Figure 1A). The thickness of the medial vessel wall in the VDN group was 114.55 ± 10.65 μm compared with the control group (76.71 ± 6.72 μm). Corylin treatment reduced this thickness to 95.82 ± 10.36 μm (Figure 1C). The calcified nodules were hardened by calcium deposits in the vessel wall in VDN rats, while corylin treatment reduced them, as assessed by Alizarin Red S staining (Figure 1B). Additionally, the quantification of calcium content in aortic tissues increased to 208.36 ± 22.67 μg/mg in VDN rats compared with the controls (110.67 ± 29.09 μg/mg), but decreased to 126.23 ± 17.48 μg/mg with corylin (Figure 1D). Similarly, alkaline phosphatase (ALP) activity was elevated in VDN‐induced aortic tissues and markedly reduced by corylin treatment (Figure 1E). Moreover, we evaluated corylin‐only treatment groups for 2 and 4 weeks, respectively, to confirm its safety profile and found that it did not cause significant effects in healthy animals. (Figure S1). These results indicated that corylin treatment attenuates aortic calcification induced in VDN‐treated rats.

**TABLE 2:** Measurement of BUN and CRE in rat serum by FUJI DRI‐CHEM SLIDES.

Group	Weeks (W)	CRE (mg/dL)	BUN (mg/dL)
CTL	W0 W4	0.4 ± 0.11 0.4 ± 0.74	15 ± 0.85 17 ± 1.02
VDN	W0 W4	0.4 ± 0.18 3.2 ± 0.69^a^	16 ± 0.63 143 ± 1.31^a^
VDN + Corylin	W0 W4	0.5 ± 0.34 1.9 ± 0.17***	16 ± 0.94 70 ± 0.57***
Corylin‐only	W0 W4	0.4 ± 0.42 0.5 ± 0.29	15 ± 1.41 18 ± 0.96

*Note:* One‐way ANOVA was used for multiple‐group comparisons. All values are presented as mean ± SEM, *n* = 6.

^a^ *p* < 0.001 compared with the untreated control group in W4.

*** *p* < 0.001 compared with the VDN‐treated group in W4.

### 3.2 Corylin Suppresses Osteogenic Differentiation and Reduces ROS Generation

**FIGURE 2:**
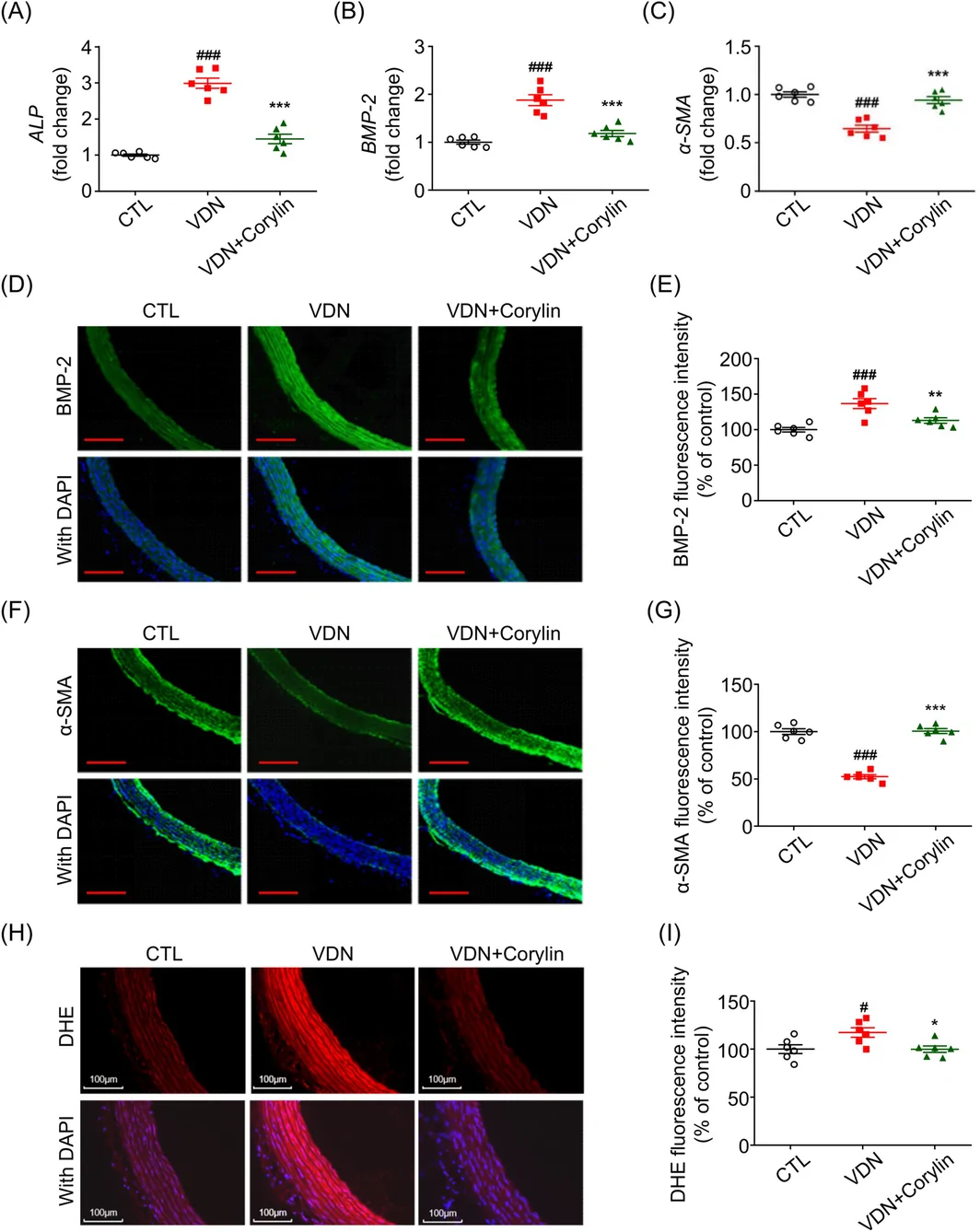
Corylin suppresses osteogenic differentiation and reduces ROS generation in VDN‐induced arterial calcification. (A, B) The expression levels of *ALP* (A) and *BMP‐2* (B) were detected by qPCR. (C) *α‐SMA* expression was measured by qPCR. (D, E) Representative images of BMP‐2 immunofluorescence and quantitative analysis. (F, G) Representative images of α‐SMA immunofluorescence and quantitative analysis. (H, I) Representative images of DHE staining and quantitative analysis. Images of nuclei counterstained with DAPI (blue) were merged. The red scale bar indicates 200 μm, and the white scale bar indicates 100 μm. One‐way ANOVA was used for multiple‐group comparisons. All values are presented as mean ± SEM, *n* = 6. #*p* < 0.05 and **p < 0.001 compared with the untreated control group. *p < 0.05, ^###^ *p* < 0.01 and ***p < 0.001 compared with the VDN‐treated group.

ALP is a widely recognised bone marker, and its expression is stimulated by bone morphogenetic protein (BMP)‐2 treatment [27]. Here, VDN administration induced *ALP* and *BMP‐2* mRNA expression, while corylin treatment suppressed (Figure 2A,B). Moreover, α‐smooth muscle actin (α‐SMA) is a cytoskeletal protein in humans and a component of the VSMC contractile unit [28]. VDN‐reduced *α‐SMA* mRNA expression was also reversed by corylin treatment (Figure 2C). Immunofluorescence staining was conducted to detect changes in the expression of BMP‐2 and α‐SMA, and to determine the effect of inhibiting osteoblastic phenotype transition after corylin treatment. Compared with that of the untreated controls, the fluorescence intensity of the osteogenic marker BMP‐2 increased significantly after induction by VDN, while corylin treatment inhibited BMP‐2 expression (Figure 2D,E). By contrast, corylin treatment markedly restored contractile protein α‐SMA fluorescence signals compared to VDN administration alone (Figure 2F,G). Thus, corylin treatment suppresses the VDN‐induced transition to an osteoblast‐like phenotype in the aorta.

ROS are reported to play an important role in osteochondrogenic phenotype switching, which is strongly associated with vascular calcification [29]. We used dihydroethidium (DHE) staining to investigate the ability of corylin to scavenge superoxide production, which was greater in the VDN‐treated group than in the control group. However, corylin treatment reduced the VDN‐induced increase in ROS levels in the aorta, as determined by DHE staining (Figure 2H,I). These results suggested that corylin scavenges ROS in vivo and ameliorates the osteogenic phenotypic transition promoted by oxidative stress in rats with VDN‐induced vascular calcification.

### 3.3 High Inorganic Phosphate‐Induced Cell Death and Apoptosis in VSMCs

**FIGURE 3:**
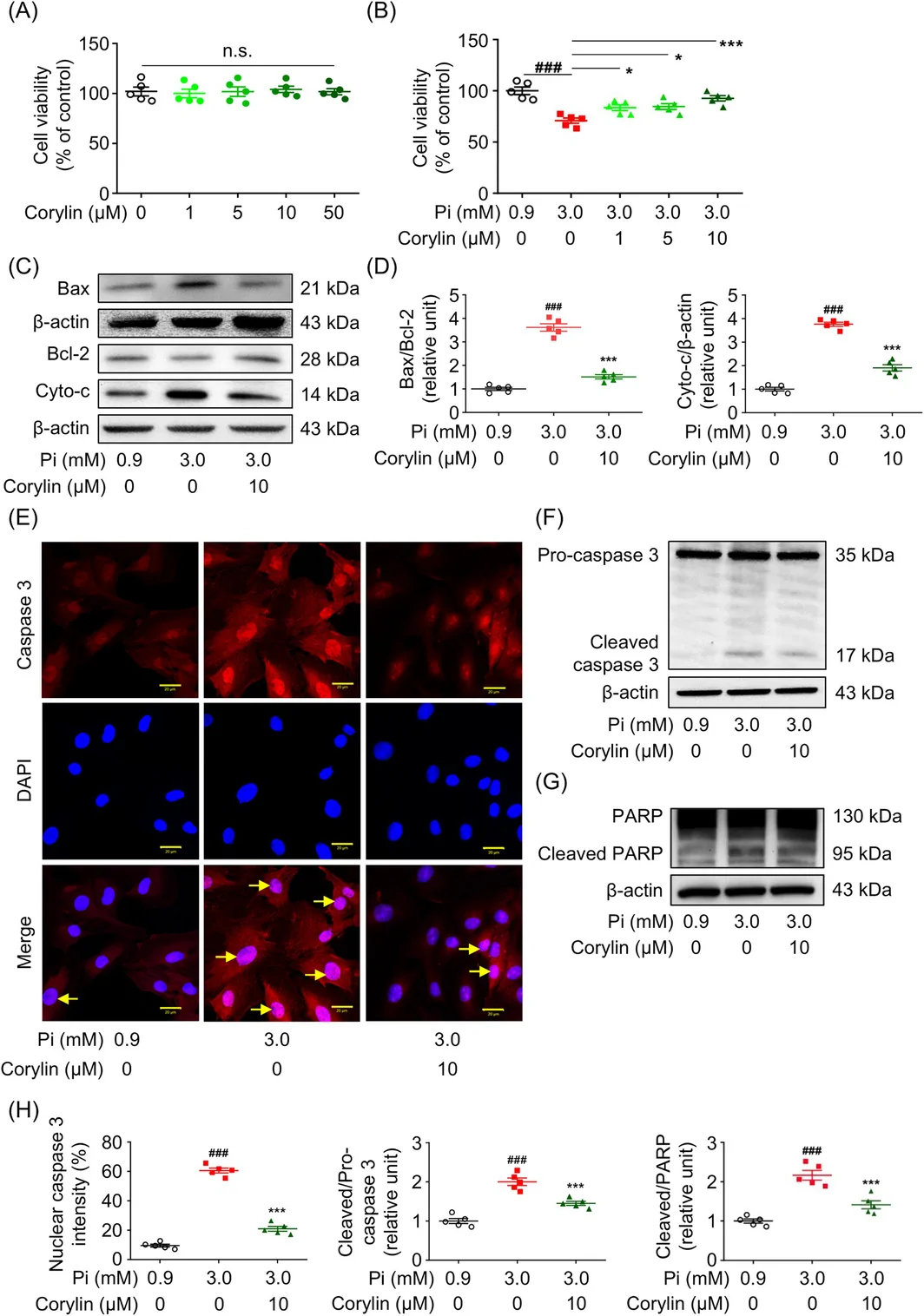
Corylin reduces high inorganic phosphate (Pi)‐induced cell death and apoptosis in rat vascular smooth muscle cells (VSMCs). (A) Cell viability after treatment with various concentrations of corylin was measured by MTT assay. (B) Cell death under high Pi conditions was determined using the MTT assay. (C, D) The protein levels of Bax, Bcl‐2, and Cyto‐c were examined by Western blotting and analysed quantitatively. (E) Representative images of activated caspase 3 immunofluorescence in the nucleus. (F, G) The protein levels of cleaved caspase 3 and PARP were determined by Western blotting. (H) Quantitative analysis of the relative nuclear caspase 3 fluorescence intensity and quantitative immunoblotting. All scale bars indicate 20 μm. One‐way ANOVA was used for multiple‐group comparisons. All values are presented as mean ± SEM, *n* = 5. Non‐significant (n.s.) compared with the cells without corylin treatment. ^###^ *p* < 0.001 compared with the group at normal Pi. *p < 0.05 and ***p < 0.001 compared with the group exposed to high Pi.

The MTT assay was used to examine the cytotoxicity of corylin at various concentrations (1, 5, 10, and 50 μM) for 24 h. Corylin had no cytotoxic effect on rat VSMCs at concentrations less than 50 μM (Figure 3A). Exposure of VSMCs to elevated phosphorus concentrations causes phosphate cytotoxicity and triggers apoptosis, which leads to vascular calcification [30]. Therefore, we first sought to evaluate the protective effect of corylin against high phosphate‐induced cytotoxicity. The cell viability decreased 70.84% ± 5.76% at high Pi concentration (3.0 mM), while pretreatment with 1, 5, and 10 μM corylin maintained cell viability at 83.53% ± 6.31%, 84.63% ± 6.40%, and 92.65% ± 5.84%, respectively, suggesting that corylin ameliorates high Pi‐induced cytotoxicity (Figure 3B). We investigated the inhibitory effect of corylin on high Pi‐induced apoptosis. The apoptosis of VSMCs occurs before the onset of calcification, and apoptosis appears to accelerate calcification [31]. High Pi‐induced apoptotic VSMCs exhibited decreased expression of Bcl‐2 and increased expression of Bax and cytochrome‐c (Cyto‐c), whereas corylin pretreatment upregulated Bcl‐2 to prevent apoptosis by blocking Cyto‐c release from mitochondria (Figure 3C,D). Moreover, immunofluorescence and Western blotting showed that corylin attenuated high Pi‐induced nuclear translocation of the downstream apoptotic effectors, activated caspase 3 and PARP (Figures 3E–H and S2). These results demonstrate that corylin protects VSMCs against high Pi‐induced cytotoxicity and apoptosis.

### 3.4 Reversal of High Pi‐Induced Calcification and Osteochondrogenic Conversion in Aortas and VSMCs

**FIGURE 4:**
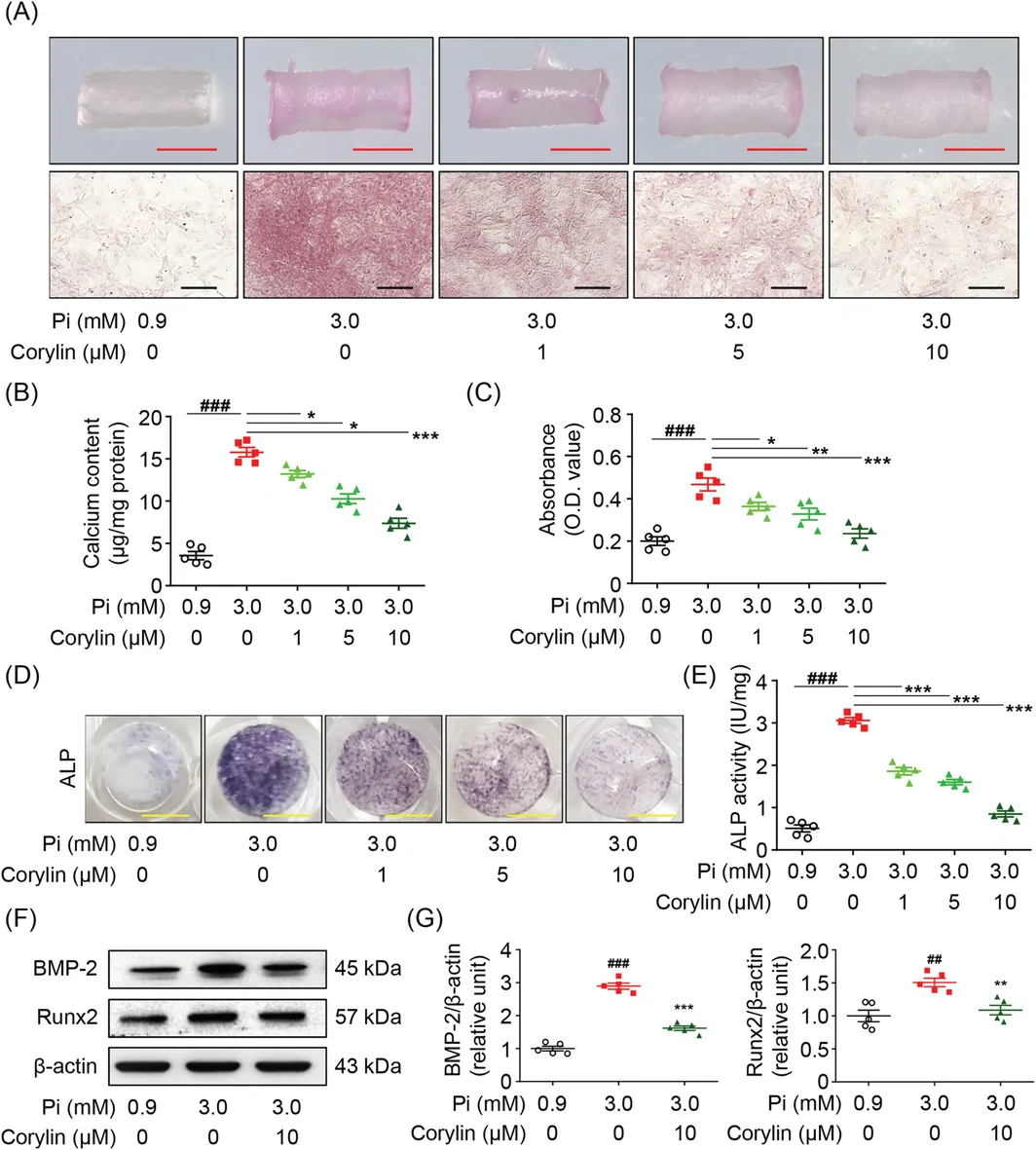
Corylin ameliorates calcification and reverses osteochondrogenic transdifferentiation induced in aortas and VSMCs with high Pi. (A) Representative images of Alizarin red S staining were used to assess calcium deposition in the thoracic aorta and in VSMCs. (B) Quantification of the calcium content in aorta homogenates. (C) Quantitative analysis of VSMCs using an Alizarin red S assay with cetylpyridinium chloride extraction. (D) Representative images of alkaline phosphatase (ALP) staining and (E) quantification of activity in the VSMCs. (F, G) The protein levels of BMP‐2 and Runx2 were quantified by Western blotting. The red scale bar indicates 2 mm, the black scale bar indicates 100 μm, and the yellow scale bar indicates 5 mm. One‐way ANOVA was used for multiple‐group comparisons. All values are presented as mean ± SEM, *n* = 5. ##*p* < 0.01 and ^###^ *p* < 0.001 compared with the group at normal Pi. *p < 0.05, **p < 0.01, and ***p < 0.001 compared with the group exposed to high Pi.

To investigate the inhibitory effect of corylin on the vascular mineralisation process further, Alizarin Red S staining assessed the high phosphate (Pi)‐induced pathological deposition of minerals in rat thoracic aortas on day 7. Rat thoracic aortas were incubated in a high Pi‐calcifying medium to follow the wide distribution of calcium, which corylin treatment significantly ameliorated. (Figure 4A). The calcium content of aortas induced by high Pi significantly increased to 15.78 ± 1.26 μg/mg compared with that of aortas in normal phosphate (3.56 ± 1.08 μg/mg). Corylin treatment at various concentrations (1, 5, and 10 μM) reduced the calcium content of high Pi‐treated aortas to 13.20 ± 0.92 μg/mg, 10.28 ± 1.29 μg/mg, and 14.93 ± 1.96 μg/mg, respectively (Figure 4B). Additionally, calcium hydroxyapatite crystals were markedly increased in high Pi‐treated VSMCs (Figure 4A, lower panel). Corylin treatment decreased high Pi‐induced calcium nodules and crystal deposition in VSMCs (Figure 4C). Macroscopic observation of alkaline phosphatase (ALP) staining also revealed that corylin reduced high Pi‐induced mineralised matrix deposition in VSMCs (Figure 4D). Furthermore, enzymatic analysis verified that high Pi increased ALP activity to 3.06 ± 0.15 IU/mg compared with 0.51 ± 0.18 IU/mg in the presence of normal Pi, which was reversed by corylin treatment. ALP activity after treatment with 1, 5, and 10 μM corylin was 1.86 ± 0.19 IU/mg, 1.60 ± 0.14 IU/mg, or 0.85 ± 0.16 IU/mg, respectively (Figure 4E). In response to high extracellular phosphate levels, VSMCs actively switch to an osteochondrogenic phenotype [32]. To determine the regulatory effect of corylin on VSMC phenotypic switching, we measured bone morphogenetic and contractile protein expression using Western blotting. The bone morphogenetic proteins BMP‐2 and Runx2 were elevated in the high Pi group, but their expression was reduced by corylin treatment (10 μM), indicating that corylin inhibited the phenotypic switch of VSMCs to osteoblast‐like characteristics (Figure 4F,G). In addition, the contractile proteins α‐SMA and SM22α expression were measured. The high Pi‐induced decreased levels of contractile proteins were restored by corylin treatment, suggesting that corylin reversed the phenotypic transition (Figure S3A,B). These results indicated that corylin decreases calcium deposition and restrains VSMC phenotypic transition from a contractile to an osteochondrogenic phenotype under high Pi stimulation.

### 3.5 Calcium Regulation and Mitochondrial Fission‐Fusion

**FIGURE 5:**
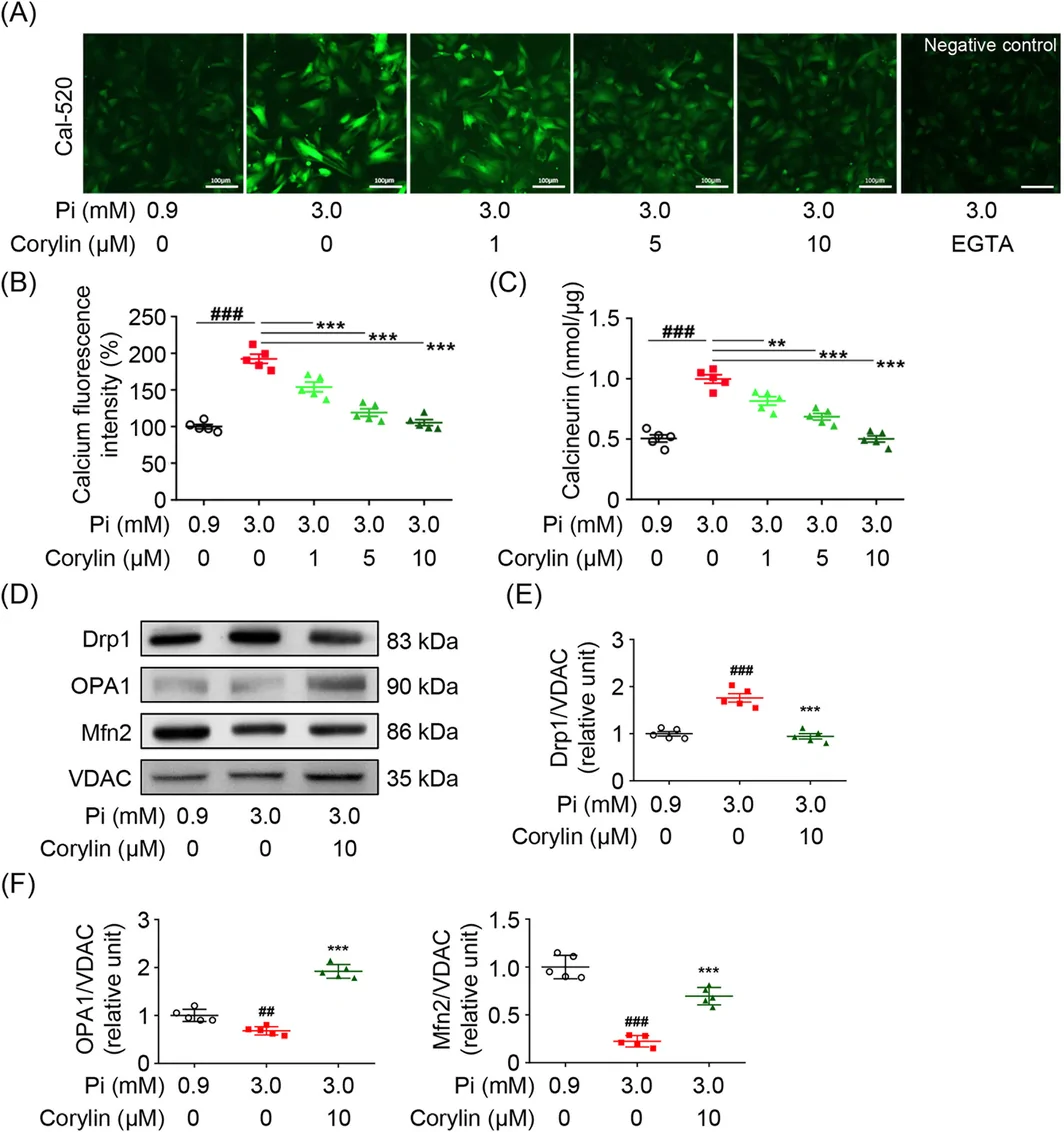
Corylin is a calcineurin inhibitor that suppresses intracellular calcium enhancement and restores mitochondrial fission‐fusion homeostasis. (A, B) Representative images of the Cal‐520 AM stain were used to detect intracellular calcium levels and for quantitative analysis. EGTA (a calcium ion chelator) as a negative control. (C) Quantitative analysis of cellular calcineurin activity by ELISA. (D–F) The levels of the mitochondrial proteins Drp1, OPA1, Mfn2, and VDAC were quantified by Western blotting. All scale bars indicate 100 μm. One‐way ANOVA was used for multiple‐group comparisons. All values are presented as mean ± SEM, *n* = 5. ^##^ *p* < 0.01 and ^###^ *p* < 0.001 compared with the group at normal Pi. **p < 0.01 and ***p < 0.001 compared with the group exposed to high Pi.

Increased intracellular calcium and subsequent oxidative stress are crucial for high Pi‐induced osteogenic differentiation and VSMC calcification [33]. Therefore, we used Cal‐520 AM calcium dye to visualise intracellular calcium in VSMCs, where high Pi significantly increased fluorescence signal. However, corylin (1, 5, and 10 μM) dose‐dependently reduced it (Figure 5A,B). Calcineurin is activated by increasing calcium concentrations in VSMCs and is a hallmark of pathological vessel remodelling processes [34]. Consistent with this activation, after high Pi treatment, calcineurin activity increased from 0.51 ± 0.07 nmol/μg in the normal Pi group to 1.00 ± 0.08 nmol/μg in the VSMCs stimulated with high Pi, whereas this increase was repressed by corylin treatment. The calcineurin activity in the VSMCs treated with 1, 5, and 10 μM corylin was 0.82 ± 0.08 nmol/μg, 0.69 ± 0.06 nmol/μg, and 0.50 ± 0.06 nmol/μg, respectively (Figure 5C). To examine mitochondrial fission and fusion, we isolated mitochondrial proteins from rat VSMCs. High Pi induced the mitochondrial fission protein Drp1 expression, but corylin treatment decreased it (Figure 5D,E). Conversely, the reduced levels of the mitochondrial fusion proteins OPA1 and Mfn2 were restored by corylin treatment (Figure 5D,F). These results suggest that corylin maintains mitochondrial fission‐fusion homeostasis and intracellular calcium balance in high Pi‐stimulated VSMCs by inhibiting calcineurin.

### 3.6 The Co‐Activation of JDP2‐Nrf2 Inhibits Oxidative Stress and VSMC Calcification

**FIGURE 6:**
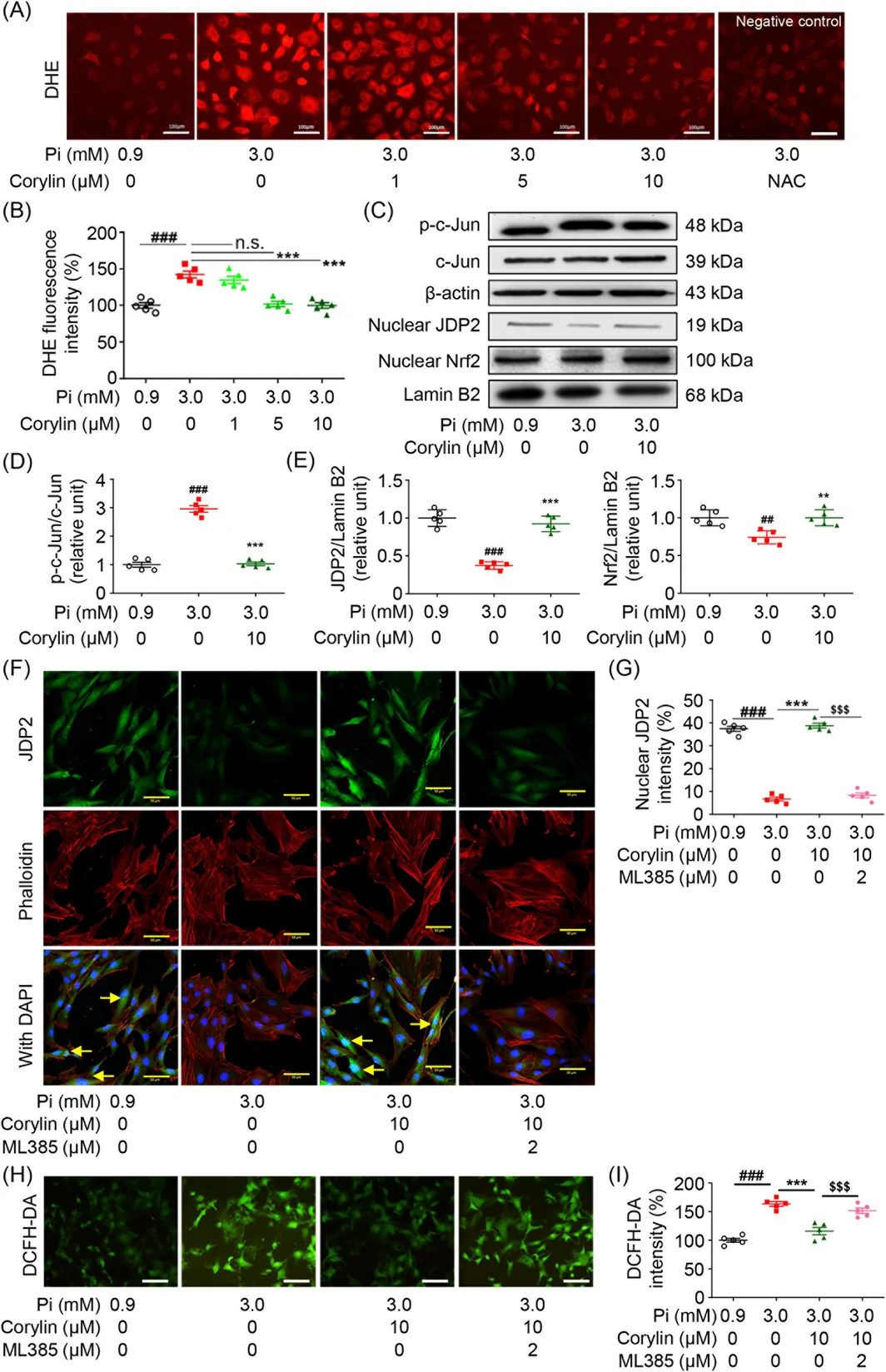
Corylin co‐activation of Jun dimerisation protein 2 (JDP2) and Nrf2 protects against oxidative stress and calcification in rat and human VSMCs. (A, B) Representative images of DHE‐stained cells used to detect ROS generation and for quantitative analysis. NAC (an antioxidant), as a negative control. (C–E) The protein levels of phospho‐c‐Jun and c‐Jun in total cell lysate (D) and the levels of the nuclear proteins JDP2, Nrf2, and lamin B2 (E) were quantified by Western blotting. (F, G) Representative images of JDP2 immunofluorescence in the nucleus and quantitative analysis. (H, I) Representative images of DCFH‐DA‐stained cells for ROS detection and quantitative analysis. The white scale bar indicates 100 μm; the yellow scale bar indicates 50 μm. One‐way ANOVA was used for multiple‐group comparisons. All values are presented as mean ± SEM, *n* = 5. #*p* < 0.01, and ^###^ *p* < 0.001 compared with the group at normal Pi. Non‐significant (n.s.), **p < 0.01 and ***p < 0.001 compared with the group exposed to high Pi. ^$$$^ *p* < 0.001 compared with high Pi + corylin treatment group.

Disruption of signalling caused by Pi overload can induce oxidative stress. Nevertheless, Pi‐induced ROS production can be reversed to serve as a potential target for pharmaceutical development [35]. The results showed that high Pi enhanced ROS production in VSMCs more than normal Pi, while DHE staining revealed that 5 and 10 μM corylin decreased high Pi‐induced ROS levels (Figure 6A,B). High ROS levels and c‐Jun phosphorylation in VSMCs cause both osteogenic differentiation and apoptosis [36]. Next, we examined the induction of phospho‐c‐Jun/c‐Jun expression in VSMCs by high Pi. Western blotting revealed that high Pi upregulated p‐c‐Jun/c‐Jun in VSMCs, which was suppressed by corylin treatment (Figure 6C,D). JDP2 is critical for basal and Nrf2‐induced transcriptional activity [11]. We used ChIP‐qPCR assays to confirm that corylin facilitates interaction between JDP2 and Nrf2. The chromatin immunoprecipitation of 10 μM corylin‐primed human VSMCs with an anti‐Nrf2 antibody showed a more robust enrichment of *Jdp2* than the nonspecific IgG control. Thus, it confirms that corylin contributes to antioxidant Nrf2 recruitment by *Jdp2* (Figure S4). To explore the antioxidant effects of corylin treatment, we analysed the expression of the superoxide dismutases. Reduced levels of cellular SOD1 and SOD2 were restored by corylin treatment (Figure S5A,B). Corylin treatment increased the levels of nuclear JDP2 and Nrf2, which were decreased in VSMCs treated with high Pi (Figure 6C,E). Immunofluorescent staining revealed that corylin treatment restored JDP2 nuclear translocation in VSMCs treated with high Pi (Figure 6F,G). Consistent with this, these effects led to reduced ROS levels, as determined by the DCFH‐DA assay; however, this reduction was blocked by the Nrf2 inhibitor (Figure 6H,I). To determine the effect of elevated JDP2 on vascular calcification, we assessed the protective effect of corylin on *Jdp2, BMP‐2*, and *α‐SMA* in human VSMCs. The qPCR results showed that *Jdp2* expression was restored by corylin after being reduced by high Pi (Figure S6A), while this effect inhibited high Pi‐induced osteogenic switching (Figure S6B,C). Notably, the Nrf2 inhibitor abrogated these effects in the corylin‐treated cells (Figures 6F–I and S6). In conclusion, we believe that corylin alleviates vascular calcification and VSMC osteogenic switching dependent on JDP2‐Nrf2‐augmented antioxidant defences and redox homeostasis.

## 4 Discussion

The increased burden of cardiovascular disease in patients with CKD is primarily driven by vascular calcification [37]. Hyperphosphatemia creates a perfect storm of risk factors for accelerated calcification, whereas low bone mass contributes to elevated calcium and phosphate levels in the blood, which are critical for initiating the progression of VSMC calcification in patients with CKD [2, 38]. There is an urgent need to develop treatment for this disease. Corylin is a major flavonoid found in *Fructus Psoralea Corylifolia*, which is used in Chinese and Ayurvedic medicine for treating nephritis and cardiovascular diseases [39]. The modern pharmacological mechanisms of corylin activity remain unclear. The present study revealed that corylin treatment ameliorates vascular calcification and reduces ROS generation induced by VDN in rats. Corylin inhibits the high Pi‐induced cell apoptosis and calcification in rat vessels and VSCMs by suppressing calcineurin activation and maintaining mitochondrial homeostasis. Furthermore, we found that the JDP2‐Nrf2 axis plays a crucial role in countering calcification in rat and human VSMCs during corylin treatment by providing antioxidant defences and decreasing ROS.

Dysregulation of calcium and phosphate metabolism is common in patients with CKD and drives vascular calcification [40]. By contrast, vessel rings from patients with CKD accumulate calcium, while elevated phosphate increases ALP activity in vessels from those patients with CKD. However, treatment with levamisole (an ALP inhibitor) alone does not block calcification [41]. Therefore, calcification in vessels from patients with CKD is most strongly associated with VSMC death resulting from calcium and phosphate deposition. The present study showed that corylin treatment reduced the tunica media thickness in the arterial wall, decreased calcium phosphate deposition, and repressed ALP activity in vessels from rats treated with VDN (Figure 1). Oxidative stress and excessive ROS contribute to the activation of specific osteochondrogenic signal transduction pathways, thereby accelerating vascular degeneration and calcification [42]. Dalfino et al. [43], reported that both BMP‐2 and 8‐oxo‐7,8‐dihydro‐2′‐deoxyguanosine levels (8‐OHdG; a marker of oxidative stress) were greater in CKD patients than in controls, and BMP‐2 levels directly correlated with 8‐OHdG serum concentrations in CKD patients. Hydrogen peroxide increases BMP‐2 expression in endothelial cells and enhances BMP‐2‐induced ALP elevation in VSMCs [44, 45]. Our present results indicate that corylin treatment suppresses the protein and gene expression of BMP‐2 and ALP by reducing ROS production in VDN‐treated rats. Moreover, corylin treatment also mitigates the remodelling of contractile VSMCs and prevents a switch to a synthetic phenotype by restoring arterial α‐SMA protein and gene expression (Figure 2). This finding is consistent with those of Chen et al., who showed that corylin treatment reduced atherosclerotic plaque areas and ROS production in ApoE‐deficient mice fed a high‐cholesterol diet [17]. To our knowledge, our present results provide the first evidence revealing the anti‐arteriosclerotic effects of corylin on modulating osteogenic transformation induced by VDN in rats.

Hyperphosphatemia is considered a decisive determinant of vascular calcification with CKD [2]. VSMCs play a critical role in initiating and progressing vascular calcification under elevated phosphate conditions by undergoing phenotype switching [5, 6]. In addition, apoptotic VSMCs can cause medial VSMC loss and degeneration, elastin breaks, and alterations in extracellular matrix composition within the medial layer of the arteries, contributing to vascular mineralisation in a uremic mouse model of CKD [46]. Moreover, pharmacological agents that inhibit apoptosis, including a caspase inhibitor (Z‐VAD‐FMK), can reduce VSMC mineralisation [47]. Our in vitro study showed that corylin acts as a preventive agent against high Pi‐induced cytotoxicity and activation of caspase 3 in VSMCs by mediating mitochondrial caspase‐dependent pathways (Figure 3). Furthermore, our study also showed that corylin treatment can alleviate high Pi‐induced mineralisation in vessels ex vivo and in VSMCs in vitro by reversing osteochondrogenic transformation (Figure 4). These data support that corylin possesses antiapoptotic and anticalcifying properties against hyperphosphatemia‐induced arteriosclerosis.

Mitochondrial dysfunction can lead to pathological VSMC phenotypic transformation induced by calcium overload and oxidative stress, further accelerating atherosclerosis progression in patients with hyperphosphatemia [33]. Calcineurin plays a crucial role in regulating VSMC development, inflammation, proliferation, and migration within the vessel walls of arteries, all of which are key hallmarks of pathological remodelling processes [34]. Esteban et al. [48], found that pharmacological inhibition of calcineurin or lentiviral delivery of calcineurin‐inhibitory peptides prevents angiotensin II‐induced aneurysm formation and restenosis in mouse models of atherosclerosis. Moreover, mitochondrial depolarisation inducers cause the translocation of the fission protein Drp1 to mitochondria. Mitochondrial depolarisation is associated with increased cytosolic calcium and activation of cytosolic calcineurin, which subsequently interacts with Drp1 [49]. We found that pharmacological inhibition of calcineurin blocked high Pi‐induced intracellular calcium release and mitochondrial fission and restored mitochondrial fusion during corylin treatment (Figure 5).

Oxidative stress plays a key role in the pathogenesis of atherosclerosis in patients with CKD, while under hyperphosphataemic conditions, the transdifferentiation of VSMCs into osteoblast‐like cells primarily causes medial arterial calcification [50]. Zhao et al. [51], and our group [20] have demonstrated calcium deposition, phenotype switching, and ROS generation in bovine and rat aortic VSMCs using a calcification model induced by β‐glycerophosphate in vitro. Pi overloading enhanced the generation of ROS and reduced nitric oxide production in bovine aortic endothelial cells, an effect that was abolished by an NADPH oxidase inhibitor [52]. The detrimental biological effects of Pi‐induced ROS production can be reversed by treatment with antioxidants or inhibitors of cytoplasmic and mitochondrial Pi transporters [36]. JDP2 uses its DNA‐binding domain as a heterodimer with Nrf2 or MafK [11]. The activation of the Nrf2‐dependent antioxidant genes and enzymes plays a crucial role in cellular defence against oxidative stress. Systemic enhancement of Nrf2 activity by various activators may be beneficial in the treatment of cardiometabolic diseases [53]. Notably, Li et al. recently became the first to demonstrate, through molecular docking analysis, that corylin binds to the Nrf2‐binding cavity of the Keap1‐Nrf2 complex via hydrogen bonds and π‐π interactions, thereby activating Nrf2 signalling and enhancing the expression of downstream antioxidant genes to protect against skin ageing [16]. Furthermore, JDP2 represses Activator Protein‐1 transcription involving competition for DNA binding and inhibition of histone acetylation and c‐Jun phosphorylation [54]. The results of our in vitro study indicated that corylin treatment upregulated *Jdp2*‐Nrf2 recruitment, reversed JDP2‐Nrf2 expression, and inhibited the phosphorylation of c‐Jun in human and rat VSMCs. This effect augmented antioxidant defence against high Pi‐induced oxidative stress and VSMC phenotypic switching (Figure 6).

Our study still has some limitations. Firstly, although an earlier study showed that estrogen inhibits VSMC osteoblastic differentiation and arterial calcification in vivo [55]; therefore, only male rats were used in this study to avoid confounding effects from female sex hormones. Secondly, the absence of a commercially available JDP2 inhibitor restricts our ability to directly evaluate JDP2 inhibition in this experimental model. Our previous studies suggest that *Jdp2* knockout embryonic fibroblasts exhibit increased resistance to oxidative stress, while impaired Nrf2/MafK activity leads to a more oxidised environment [11]. Additionally, Nrf2‐JDP2 complexes regulate Slc7a11 expression to control ROS homeostasis and ROS‐induced cell death in cerebellum granule cell progenitors from *Jdp2* knockout mice [56, 57]. Future studies using *Jdp2* knockout mice are needed to better define how corylin and JDP2 regulate vascular calcification, providing stronger evidence than siRNA knockdown approaches. Third, due to technical constraints and restricted access to patient specimens, the clinical relevance of our results requires further validation in human cohorts.

## 5 Conclusion

**FIGURE 7:**
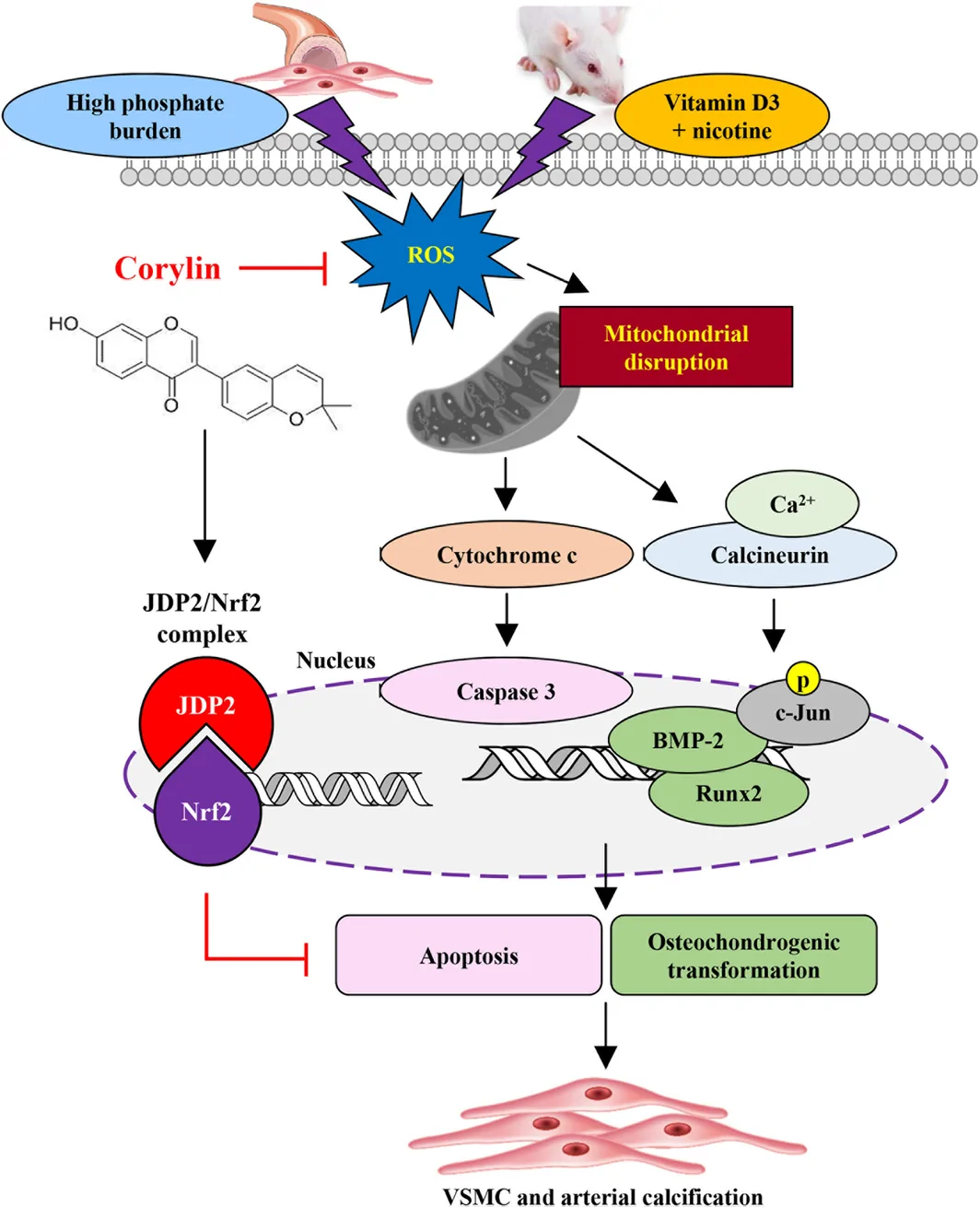
Schematic illustration of how corylin protects vascular smooth muscle cells and alleviates vascular calcification via activating the JDP2‐Nrf2 axis to maintain ROS homeostasis.

In summary, we demonstrate that corylin has anti‐arteriosclerotic and vasculoprotective effects by suppressing VSMC phenotype switching and mineralisation and reducing oxidative stress through JDP2‐Nrf2 activation, both in vivo and in vitro (Figure 7). Thus, our preclinical research reveals that corylin is a promising antioxidant recommended for daily intake to help mitigate vascular calcification in CKD patients.

## Author Contributions

**Kazunari K. Yokoyama:** writing – original draft, data curation. **Shang‐En Huang:** writing – original draft, writing – review and editing, investigation, data curation. **Yu‐Hsin Tseng:** methodology, project administration, writing – review and editing. **Kai‐Fang Hu:** investigation, data curation. **Zen‐Kong Dai:** data curation. **Meng‐Xuan Lin:** investigation. **Jong‐Hau Hsu:** writing – review and editing, conceptualization, funding acquisition. **Jwu‐Lai Yeh:** funding acquisition, conceptualization, writing – review and editing. **Chih‐Le Lin:** data curation, investigation. **Bin‐Nan Wu:** data curation.

## Funding

Our work was supported by grants from NSTC 112‐2320‐B‐037‐019‐MY3 (JL Yeh), NSTC 115‐2320‐B‐992‐001 (SE Huang), and MOST 111‐2314‐B‐037‐075‐MY3 (JH Hsu) from the National Science and Technology Council; and was supported by KMU‐TB114009‐2 (JL Yeh) and KMU‐DK(N)115004 (JL Yeh) from Kaohsiung Medical University and KMUH 114‐4R48 (JH Hsu) from Kaohsiung Medical University Chung‐Ho Memorial Hospital.

## Conflicts of Interest

The authors declare no conflicts of interest.

## Supporting information


**Figure S1:** The feasibility assessment of corylin treatment in rats. (A) Representative histological images with H&E staining. (B) The histological assay of calcium by Alizarin red S staining. (C) The thickness of the tunica media was quantified using AxioVision software. (D) Quantification of the calcium content and (E) ALP activity in the thoracic aorta homogenates. All scale bars indicate 100 μm. One‐way ANOVA was used for multiple‐group comparisons. All values are presented as mean ± SEM, *n* = 6. Non‐significant (n.s.) compared with the untreated control group.

**Figure S2:** Corylin suppressed high Pi‐induced PARP cleavage in human VSMCs. (A, B) The expression of PARP cleavage in cells was quantified by Western blotting. One‐way ANOVA was used for multiple‐group comparisons. All values are presented as mean ± SEM, *n* = 5. ^###^ *p* < 0.01 compared with the group at normal Pi. ***p < 0.01 compared with the group exposed to high Pi.

**Figure S3:** Corylin restores contractile protein phenotypic variation in rat VSMCs treated with high Pi. (A, B) The expression of α‐SMA and SM22α in VSMCs was quantified by Western blotting. One‐way ANOVA was used for multiple‐group comparisons. All values are presented as mean ± SEM, *n* = 5. ##*p* < 0.01 compared with the group at normal Pi. *p < 0.05 and **p < 0.01 compared with the group exposed to high Pi.

**Figure S4:** jcmm71331‐sup‐0004‐FigureS4.tif. *Jdp2* potentiates the transcriptional activation of Nrf2 in corylin‐primed human aortic SMCs. Chromatin immunoprecipitation of 10 μM corylin‐primed human VSMCs, as detected by ChIP‐qPCR assay. IgG was used as a negative control. An unpaired Student's *t*‐test for two‐group comparisons. All values are presented as mean ± SEM, *n* = 5. ***p < 0.001 compared to the IgG‐mediated precipitation.

**Figure S5:** Corylin re‐establishes antioxidant‐related superoxide dismutase activity against oxidative stress in the high Pi‐treated VSMCs. (A, B) The expression of SOD1 and SOD2 in rat VSMCs was quantified by Western blotting. One‐way ANOVA was used for multiple‐group comparisons. All values are presented as mean ± SEM, *n* = 5. #*p* < 0.05 and ^###^ *p* < 0.001 compared with the group at normal Pi. ***p < 0.001 compared with the group exposed to high Pi.

**Figure S6:** Corylin restores *Jdp2* levels to protect against osteogenic switching in human VSMCs exposed to high Pi, reversed by ML385. (A–C) The expression levels of *Jdp2* (A), *BMP‐2* (B), and *α‐SMA* (C) were detected by qPCR. One‐way ANOVA was used for multiple‐group comparisons. All values are presented as mean ± SEM, *n* = 5. ^###^ *p* < 0.001 compared with the group at normal Pi. **p < 0.01 and ***p < 0.001 compared with the group exposed to high Pi. ^$$^ *p* < 0.01 and ^$$$^ *p* < 0.001 compared with high Pi + corylin treatment group.

## Data Availability

The data that support the findings of this study are available from the corresponding author upon reasonable request.
